# Thyroglobulin fluctuations in patients with iodine-refractory differentiated thyroid carcinoma on lenvatinib treatment – initial experience

**DOI:** 10.1038/srep28081

**Published:** 2016-06-16

**Authors:** R. A. Werner, K. Lückerath, J. S. Schmid, T. Higuchi, M. C. Kreissl, I. Grelle, C. Reiners, A. K. Buck, C. Lapa

**Affiliations:** 1 Department of Nuclear Medicine, University Hospital Würzburg, Würzburg, Germany; 2 Comprehensive Heart Failure Center, University Hospital Würzburg, Würzburg, Germany; 3 Department of Nuclear Medicine, Hospital Augsburg, Augsburg, Germany

## Abstract

Tyrosine kinase inhibitors (TKI) have shown clinical effectiveness in iodine-refractory differentiated thyroid cancer (DTC). The corresponding role of serum thyroglobulin (Tg) in iodine-refractory DTC has not been investigated yet. 9 patients (3 female, 61 ± 8y) with progressive iodine-refractory DTC starting on lenvatinib were considered. Tumor restaging was performed every 2–3 months including contrast-enhanced computed tomography (CT, RECIST 1.1). Serum Tg was measured and compared to imaging findings. After treatment initiation, serum Tg levels dropped in all patients with a median reduction of 86.2%. During long-term follow-up (median, 25.2 months), fluctuations in Tg could be observed in 8/9 subjects. According to RECIST, 6/9 subjects achieved a partial response or stable disease with the remaining 3/9 experiencing progressive disease (2/3 with Tg levels rising above baseline). All of the patients with disease progression presented with a preceding continuous rise in serum Tg, whereas tumor marker oscillations in the subjects with controlled disease were only intermittent. Initiation of lenvatinib in iodine-refractory DTC patients is associated with a significant reduction in serum Tg levels as a marker of treatment response. In the course of treatment, transient Tg oscillations are a frequent phenomenon that may not necessarily reflect morphologic tumor progression.

In the past decade, the incidence of thyroid cancer has increased faster than that of any other malignancy with differentiated thyroid cancer (DTC) accounting for >90% of all cases^1^,^2^. Whereas overall prognosis is extremely good with most DTC patients not dying from their disease^3^, 10-year survival rates have been reported to be as low as 10% in patients with radioiodine-resistant/-refractory disease^4^,^5^. As treatment options in systemic radioiodine-refractory disease, tyrosine kinase inhibitors (TKI) such as sorafenib, vandetanib and pazopanib have shown clinical effectiveness^6^,^7^,^8^,^9^,^10^. However, to date, sorafenib and lenvatinib are the only compounds which demonstrated efficacy in dedicated multicenter phase III trials. The DECISION trial using sorafenib showed a significant improvement in progression-free survival (PFS) of 10.8 months (vs. 5.8 months in the placebo group)^6^. In the SELECT trial, lenvatinib could demonstrate significantly increased PFS in patients with progressive radioiodine-refractory DTC^11^. In comparison to sorafenib, lenvatinib even represented the most active agent with a better tumor response rate and an improved PFS of 18.3 months^12^. Based on these results, both drugs have been approved by the FDA for the treatment of locally recurrent or metastatic, progressive DTC that no longer responds to radioactive iodine treatment.

In order to assess effectiveness of TKI treatment, morphologic tumor measurement based on computed tomography is routinely used to monitor patients^13^,^14^. The role of serum thyroglobulin (Tg) in this scenario is not entirely clear: Whereas short-term rises of serum tumor markers (calcitonin, carcinoembryonic antigen [CEA]) not reflecting tumor progression have been reported in patients with medullary thyroid carcinoma (MTC) during TKI treatment^15^, the corresponding kinetics of Tg in radioiodine-refractory DTC patients have not been investigated yet. Given the rising importance and more widespread clinical use of TKI in the treatment of radioiodine-refractory DTC outside the setting of controlled clinical trials, knowledge of serum tumor marker kinetics and their association with response to treatment is urgently needed and might allow for the choice of the best time point to order imaging tests or modify treatment due to tumor progression.

In this pilot study we assessed the time course of serum Tg levels and their correlation to imaging findings (i.e. to tumor measurements according to RECIST) in radioiodine-refractory DTC patients treated with lenvatinib.

## Methods

Between August 2012 and October 2015, 9 patients (6 males, 3 females; mean age, 61 ± 8y) started on oral lenvatinib (24 mg (n = 7) or 20 mg (n = 2) daily) due to progressive, radioiodine-refractory DTC at the University Hospital of Würzburg, Germany. All of the subjects enrolled were on thyroid hormone replacement therapy with low to suppressed thyroid stimulating hormone levels and presented with an Eastern Cooperative Oncology Group (ECOG) performance status ≤2.

All patients gave written informed consent to the therapeutic and diagnostic procedures. Since our study comprises a retrospective analysis of routinely acquired data, the local ethic committee waives the need for further approval.

### Tumor response assessment

Tumor response was assessed according to Response Evaluation Criteria in Solid Tumors (RECIST) 1.1 based on routine computed tomography (CT) performed every 2–3 months^14^. RECIST measurements were confirmed by both an attending nuclear medicine physician and radiologist. All scans were performed using a 64-slice spiral CT (SOMATOM Sensation 64, Siemens Medical Solutions, Erlangen, Germany) with intravenous contrast enhancement (care dose modulation with a quality reference of 210 mAs, 120 kV, a 512 × 512 matrix, 5 mm slice thickness), covering the base of the skull to the proximal thighs.

### Tumor marker thyroglobulin

Serum Tg levels (ng/ml) were measured at baseline and at each outpatient visit using dedicated immunoradiometric assays (Thermofisher Scientific, Henningsdorf, Germany) with an analytical sensitivity of 0.08 ng/ml and a functional sensitivity of 0.2 ng/ml. An immunoradiometric recovery assay (Thermofisher Scientific, Henningsdorf, Germany) was used to exclude potential interference of thyroglobulin antibodies.

### Analysis and statistics

Most of the observations described are of descriptive nature. Statistical analyses were performed using PASW Statistics software (version 22.0; SPSS, Inc. Chicago, IL, USA). Quantitative values were expressed as mean (±standard deviation) or median and range as appropriate.

## Results

### Patients

At baseline all patients presented with progressive metastatic iodine-refractory DTC. 2/9 patients suffered from papillary, 5/9 subjects from follicular and the remaining 2/9 subjects from oncocytic thyroid cancer. All patients had undergone various therapies including surgery (n = 9/9), radioiodine treatment (n = 9/9), external beam radiation (n = 4/9), sorafenib (n = 2/9), and peptide receptor radionuclide therapy (n = 1/9).

Metastatic sites included lung (n = 9/9), lymph nodes (n = 8/9), bone (n = 5/9) and liver (n = 2/9). Median follow-up of subjects was 25.2 months (range, 2–37 months). Detailed patient characteristics’ are given in Table 1.

### Tumor response

All subjects achieved clinical benefit from lenvatinib with initial tumor responses to treatment in all cases (median reduction in size of target lesions, 38.7%; range, 17–86%). According to RECIST 1.1., 6/9 (66.6%) patients achieved partial response, the remaining 3/9 (33.3%; patients #2, #4 and #6) stable disease (SD) as best response. The nadir of tumor sizes could be recorded after a median of 8.0 months (range, 2–27 months).

At present, after a mean follow up of 19 months, only 3/9 patients have escaped from treatment: patients #4, #8 and #9 progressed 3 (patient #4), 22 (patient #8) and 33 months (patient #9) after initiation of lenvatinib, respectively. Tumor progression was defined by an increase in target lesions >20% in 1/3 patients (patient #9) as well as the occurrence of new lesions (patients #4, #8 and #9).

Two of the patients with PD died from their cancer after 4 (patient #4) and 36 months (patient #8), respectively. All other patients are still alive as of November 1^st^, 2015.

During follow-up, fluctuations in tumor sizes could be observed in 5/9 subjects. Increases in RECIST measurements after initial response were <20% in 3/5 cases, the remainder (patients #4 and #9) presented with an increase of 65% and 45%, respectively.

### Thyroglobulin kinetics

Prior to treatment all patients had elevated Tg levels with median baseline values of 6352 ng/ml (range, 7–24495 ng/ml). An initial decrease of Tg (at first restaging) was recorded in all subjects with a median redcution of 86.2% (range, 73–99%). The nadir of Tg was reached after a median of 5.8 months (range, 2–14 months). In 4/9 patients (patients #2, #4, #7 and #9) the nadir of both Tg levels and RECIST measurements matched. In the remaining 5 patients the nadir of Tg preceded RECIST measurements by a median of 11.2 months (range, 4–22 months).

**Figure 1:**
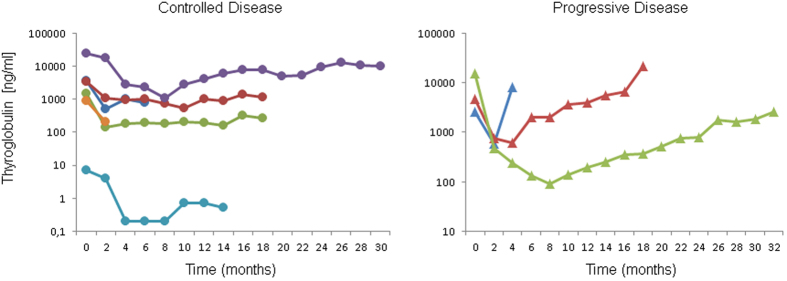
Time course of thyroglobulin (Tg) in patients with controlled (stable disease or partial response; left) or progressive disease (right). All patients display an initial decline in Tg after initiation of treatment with lenvatinib. Whereas the patients with long-term disease control present with transient fluctuations in Tg levels (left) over time, Tg rises (continuously) in the subjects with truly progressive disease (right).

Thereafter, all subjects with controlled disease exhibited mild alterations in Tg levels; however, serum marker levels in those patients never increased to baseline values (Fig. 1, left). In contrast, Tg levels increased and exceeded baseline levels in 2/3 patients with PD (patients #4 and #8). Importantly, continuously rising serum marker during follow-up (at least at 4 subsequent visits) was a precursor of tumor progression in 2/3 patients (patients #8 and #9; Fig. 1, right).

Moreover, defining an early biochemical response from the baseline Tg value after 4 weeks of lenvatinib administration, the SD/PR group demonstrated a drop of 65.5% (range, 26.0–90.3%). Interestingly, the remaining patients suffering from disease progression during follow-up even revealed a higher initial drop of Tg levels of 86.2% (range, 77.4–96.9%). The exact Tg levels prior to and after lenvatinib initiation are available for each individual patient in supplementary Table 1.

### Influence of lenvatinib dose on Tg levels

7/9 patients started TKI treatment with a daily dose of 24 mg, the remaining 2/9 subjects (patients #2 and #3) received 20 mg. In the course of treatment, the daily intake of lenvatinib was reduced from 24 mg to 20 mg in 1/9 (patient #5), to 14 mg in 4/9 (patients #2 and #3; patients #8 and #9 after a previous reduction from 24 mg to 20 mg) and to 10 mg in 1/9 patients (patient #6 after prior reduction to 20 mg and 14 mg, respectively) due to adverse events like diarrhea or hypertension. De-escalation was initiated after a median lenvatinib treatment of 4.7 months (range, 1–8 months). A reduction from 24 mg to 20 mg was associated with a rise in Tg levels in 1 case (patient #5), whereas in the remaining 3 patients (patients #6, #8 and #9), Tg levels further dropped despite reduced daily intake of lenvatinib. In these 3 patients, a further reduction to 14 mg translated to intermittent increases in Tg values in 2 patients (patients #8 and #9). In contrast, patient #6 demonstrated stable Tg levels after dose reduction, which, after changing from 14 mg to 10 mg of lenvatinib, were further slightly decreased. Patients #2 and #3 who both initially received a daily dose of 20 mg experienced a further drop (patient #2) and a slight rise (patient #3) in Tg levels.

**Figure 2:**
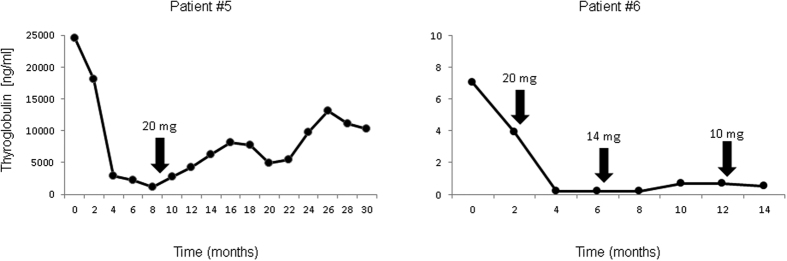
Example of the influence of dose reductions of lenvatinib on serum thyroglobulin levels. Whereas patient #5 (left) exhibited rising Tg levels after reducing the dose from 24 mg to 20 mg, tumor marker levels decreased or remained stable after several reductions from 24 mg to 10 mg in patient #6 (right). On computed tomography, both patients presented with a partial response (partient #5) and stable disease (patient #6) throughout follow-up.

Dose reductions were not associated with morphologic disease progression in any patient. Two examples of Tg oscillations after dose reduction (patients #5 and #6) are given in Fig. 2.

## Discussion

The clinical value of Tg as tumor marker in the management of DTC is well accepted^16^. However, therapy with tyrosine kinase inhibitors for medullary thyroid cancer is associated with an initial rapid decline in tumor marker levels and a subsequent phase of oscillation not necessarily indicating relapse, resistance to treatment or progressive disease.

This pilot study is the first report on the potential association between serum Tg levels and anatomic tumor changes in DTC patients treated with lenvatinib. In our cohort enrolling iodine-refractory patients with progressive disease, we could observe an initial decline in Tg levels in all patients with a median drop to a nadir of 86.2%. In parallel, all patients experienced in part morphological responses according to RECIST, resulting in a partial response or stable disease. Of note, the nadir of both parameters matched in less than 50% of the cases (with changes in Tg levels preceding anatomic responses in the remainder), thus underlining different kinetics and confounding factors of both follow-up tools. Despite serum Tg fluctuations in 8/9 patients in the subsequent course of treatment, this response was maintained in 6/9 subjects enrolled. Of note, all cases with controlled disease presented with only short-term fluctuations in Tg, whereas 2/3 patients with true PD experienced a preceding continuous rise of tumor marker levels (>4 rises in a row) spiking above baseline levels in one of these, a finding which could also be observed in medullary thyroid cancer undergoing TKI treatment with vandetanib^17^. Interestingly, in our patient cohort, PD was associated with the emergence of new metastases (3/3 subjects with PD) whereas the known tumor lesions were still largely controlled by lenvatinib in 2/3 cases. While it is too early to draw any conclusions on underlying mechanisms of tumor escape, this finding should be further investigated in future studies. Our findings are in line with previously published reports on short-term fluctuations in tumor marker levels in patients treated with TKI, adding evidence that changes in tumor markers not always reflect changes in tumor size^15^,^18^,^19^. Potentially, transient oscillations in serum Tg levels are the equivalent of short-term changes in the metabolic activity of tumor that do not translate into anatomic growth. This hypothesis is supported by the fact that in 2/3 cases with disease progression, a continuous rise in serum Tg levels (at least at 4 subsequent visits) was recorded before progressive disease could be visualized on CT. The notion that functional changes precede morphologic tumor responses to treatment has already been demonstrated in other tumor entities including adenocarcinoma of the oesophagogastric junction or gastrointestinal stromal tumor^20^,^21^.

In this study, we could also assess the effect of (subsequent) dose reductions on tumor marker levels. Whereas a reduction from 24 mg to 20 mg was associated with a drop in Tg levels or RECIST measurements in 3/4 cases, a reduction from 20 mg to 14 mg translated to intermittent increases in serum tumor marker values in 3/5 patients (without changes in RECIST in any of the patients). Therefore, even an increase in Tg after reduction of the daily oral dose of TKI can be observed before taking further diagnostic or therapeutic steps. Given the increasing importance and more widespread use of this class of drugs (e.g. due to FDA approval for lenvatinib and sorafenib), the rising number of long-term treatments and the growing numbers of RET- or other kinase-directed medications, our findings may help the clinician to discern the optimal time point for further work-up and, potentially, a change in treatment management (e.g. prescription of another TKI).

This study has some limitations. First, only a small number of patients could be included, thereby limiting statistical power of analyses. Second, this is a retrospective analysis and a prospective approach is necessary to strengthen our preliminary findings. Finally, the effect of short-term drug discontinuation was not assessed. However, to the best of our knowledge, this is the first report of a patient cohort exclusively treated with lenvatinib with a median follow-up period of 2 years.

In summary, in iodine-refractory DTC patients undergoing treatment with lenvanitib, serum Tg fluctuations are a frequent phenomenon that do not necessarily reflect morphologic tumor alterations in these patients, especially shortly after lenvatinib dose reductions. Whereas patients with truly controlled disease present with oscillating tumor markers after an initial nadir without morphologic tumor progression, patients with true PD demonstrate a continuous rise in Tg. Larger prospective studies are needed to fully elucidate the association between tumor marker levels and tumor biology.

## Conclusions

Initiation of lenvatinib in iodine-refractory DTC patients is associated with a significant reduction in serum Tg levels as a marker of treatment response. In the course of treatment, transient Tg oscillations are a frequent phenomenon that may not necessarily reflect morphologic tumor progression. However, a continuous rise in Tg levels should raise the concern for tumor progression.

## Additional Information

**How to cite this article**: Werner, R.A. *et al*. Thyroglobulin fluctuations in patients with iodine-refractory differentiated thyroid carcinoma on lenvatinib treatment – initial experience. *Sci. Rep.* **6**, 28081; doi: 10.1038/srep28081 (2016).

## Supplementary Material

Supplementary Information

## Figures and Tables

**Table 1:** Detailed patients’ characteristics.

	**Sex**	**Age (y)**	**WHO/ECOG**	**Disease duration (y)**	**DTC type**	**Extent of disease**	**Metastatic sites**	**Prior therapy**	**Long-term TKI response**
1	f	47	1	16	oncocytic	metastatic	LN, bone, liver, lung	surgery, RIT, PRRT	PR
2	m	52	0	10	oncocytic	metastatic	LN, bone, lung	surgery, RIT, RTx	SD
3	f	70	0	16	follicular	metastatic	LN, bone, lung	surgery, RIT	PR
4	f	64	0	11	follicular	metastatic	LN, liver, lung	surgery, RIT	PD
5	m	57	0	13	follicular	metastatic	LN, bone, lung	surgery, RIT, RTx	PR
6	m	62	2	8	papillary	metastatic	lung	surgery, RIT, sorafenib	SD
7	m	69	1	13	papillary	metastatic	LN, bone, lung	surgery, RIT	PR
8	m	68	0	21	follicular	metastatic	LN, lung	surgery, RIT, RTx	PD
9	m	64	0	6	follicular	metastatic	LN, lung	surgery, RIT, RTx, sorafenib	PD

DTC = differentiated thyroid cancer, ECOG = Eastern Cooperative Oncology Group, f = female, LN = lymph node, m = male, PD = progressive disease, PR = partial response, PRRT = peptide receptor radionuclide therapy, RIT = radioiodine therapy, RTx = external radiation therapy, SD = stable disease, TKI = tyrosine kinase inhibitor, WHO = World Health Organization.
