# Supplementary material for: Thyroglobulin fluctuations in patients with iodine-refractory differentiated thyroid carcinoma on lenvatinib treatment – initial experience
Source: Sci Rep. 2016 Jun 16;6:28081. doi: 10.1038/srep28081 (PMC4910099; doi:10.1038/srep28081)
Supplement: Supplementary Information [file srep28081-s1.pdf]

Thyroglobulin fluctuations in patients with iodine-refractory differentiated thyroid carcinoma on lenvatinib treatment – initial experience

Werner RA, MD[1,2], Lückerath K, PhD[1], Schmid JS, MD[1], Higuchi T, MD, PhD[1,2], Kreissl MC[1,3], Grelle I[1], Reiners C, MD[1], Buck AK, MD[1,2], Lapa C, MD[1]

Supplementary Table 1: Individual serum thyroglobulin (Tg) levels before and 4weeks after lenvatinib initiation

	Sex	Age (y)	DTC type	Extent of disease	Tg before TKI initiation (g/ml)	Tg after TKI initiation (g/ml)	Initial change (%)	Initial RECIST response	Long-term TKI response
**1**	f	47	oncocytic	metastatic	3654	506	-86.2	SD	PR
**2**	m	52	oncocytic	metastatic	3456	1094	-68.4	SD	SD
**3**	f	70	follicular	metastatic	1502	145	-90.3	PR	PR
**4**	f	64	follicular	metastatic	2550.7	577	-77.4	SD	PD
**5**	m	57	follicular	metastatic	24495	18115	-26.0	SD	PR
**6**	m	62	papillary	metastatic	7.0	3.9	-44.3	SD	SD
**7**	m	69	papillary	metastatic	931.7	207	-77.8	PR	PR
**8**	m	68	follicular	metastatic	4770	755	-84.2	PR	PD
**9**	m	64	follicular	metastatic	15800	483	-97.0	PR	PD

DTC = differentiated thyroid cancer, f = female, m = male, PD = progressive disease, PR = partial response, RECIST = Response Evaluation Criteria in Solid Tumors, SD = stable disease, Tg = thyroglobulin, TKI = tyrosine kinase inhibitor
